# Understanding Post-Translational Modifications in Porcine Reproductive and Respiratory Syndrome Virus Infection

**DOI:** 10.3390/vetsci11120654

**Published:** 2024-12-16

**Authors:** Xiaoyong Chen, Jianlong Zhang

**Affiliations:** 1 Xingzhi College, Zhejiang Normal University, Lanxi 321100, China; 2 Pingliang Vocational and Technical College, Pingliang 744000, China; jianlongz@163.com

**Keywords:** porcine reproductive and respiratory syndrome virus, post-translational modification, virus–host interaction

## Abstract

Porcine reproductive and respiratory syndrome virus (PRRSV) is a highly contagious virus affecting pigs with significant impacts to the swine industry worldwide. This review provides a comprehensive understanding of post-translational modifications (PTMs) associated with PRRSV infection. We discuss the various types of PTMs, including phosphorylation, ubiquitination, SUMoylation, acetylation, glycosylation, palmitoylation, and lactylation, that occur during PRRSV infection. We emphasize how these modifications affect the function and activity of viral proteins, thereby influencing virus replication, assembly, and egress. Additionally, we delve into the host cellular responses triggered by PRRSV, particularly the PTMs that regulate host signaling pathways and immune responses. Furthermore, we summarize the current understandings of how PTMs facilitate the ability of virus to evade the host immune system, enabling it to establish persistent infections. Finally, we address the implications of these modifications in the development of novel antiviral strategies and the potential for exploiting PTMs as therapeutic targets. This review highlights the significance of PTMs in shaping viral pathogenicity and host antiviral mechanisms and provides valuable insights for future research aimed at developing effective interventions against PRRSV infections.

## Simple Summary

Porcine reproductive and respiratory syndrome virus (PRRSV) poses a significant threat to the global swine industry, causing severe economic losses. A critical yet understudied aspect of PRRSV infection is the role of post-translational modifications (PTMs), which can profoundly affect viral protein function and host–virus interactions. To address this gap, this review aims to understand the PTMs in PRRSV infections. By summarizing the latest advances in PTMs under PRRSV infection, this review identifies and characterizes various PTMs in PRRSV-infected cells, revealing that PTMs like phosphorylation and ubiquitination play crucial roles in modulating viral protein activity and stability. Collectively, understanding PTMs in PRRSV infection not only advances our knowledge of viral pathogenesis but also holds promise for the development of innovative therapies and vaccines.

## 1. Introduction

**Figure 1:**
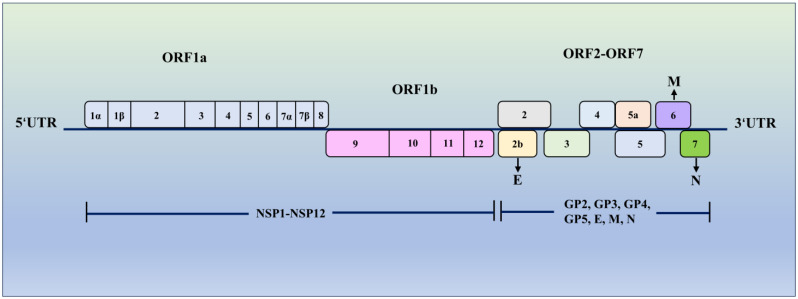
The PPRSV genome structure. The genome of PPRSV consists of several ORFs, including ORF1a, ORF1b, and ORF2-7. ORF1a expresses proteins NSP1-8 and ORF1b encodes proteins NSP9-12. ORF2-7 express glycoproteins 2-5, as well as envelope (E), membrane (M), and nucleocapsid (N) proteins.

Porcine reproductive and respiratory syndrome virus (PRRSV) belongs to the family *Arteriviridae*, with a single-stranded and positive-sense RNA of approximately 15 kilobases in length [1]. The PRRSV genome contains several ORFs, including ORF1a and ORF1b that encode nonstructural proteins (NSP) 1-12, which are involved in various aspects of virus replication and pathogenesis. For example, NSP9, an RNA-dependent RNA polymerase, modulates viral replication through its interaction with host proteins, such as DEAD-box RNA helicase 5 (DDX5) and zinc finger antiviral protein (ZAP) [2]. NSP10 encodes an RNA helicase that catalyzes the synthesis of sub-genomic mRNA (sgmRNA) and may additionally aid in the resolution of RNA secondary structures during the replication process [3]. ORF2-ORF7 encode structural proteins such as glycoproteins (GP2, GP3, GP4, and GP5) as well as the envelope (E), membrane (M), and nucleocapsid (N) proteins (Figure 1) [4]. These structural proteins play vital roles in virus assembly and the infection of host cells. The N protein has the ability to form homodimers, which are involved in binding to the RNA genome and facilitating the assembly of viral particles. In addition, N could interact with multiple host proteins to modulate viral evasion and immune responses [5]. The E protein, a nonglycosylated structural protein, is not essential for virus particle assembly but its deletion prevents the production of virus particles [6].

PRRSV poses significant threats to the swine industry worldwide, causing a range of clinical manifestations that impact both animal health and production [7]. In sows, PRRSV infection can result in reproductive failure, manifesting as abortions, stillbirths, and the birth of weak or unviable piglets [8]. In growing pigs and piglets, PRRSV often causes respiratory distress, characterized by coughing, dyspnea, and increased respiratory rates [9,10]. PRRSV outbreaks often lead to trade restrictions and quarantines, disrupting supply chains and markets and causing significant financial losses. Currently, several strategies are employed to mitigate the spread and impact of PRRSV. Firstly, biosecurity measures play a crucial role. These include strict protocols for animal movement, the thorough cleaning and disinfection of facilities, and the isolation of sick animals [11]. By reducing the introduction and spread of the virus within and between farms, biosecurity helps to maintain herd health and prevent outbreaks. Vaccines are available and are used widely, aiming to induce immunity and reduce the severity of clinical signs in infected animals [12]. However, it is important to note that vaccines may not provide complete protection, especially against newly emerging strains of the virus [13,14]. Therefore, regular updates to vaccines and vaccination strategies are necessary to ensure their continued effectiveness. In addition, surveillance and monitoring are essential components of PRRSV control [15]. The regular testing of animals for the presence of the virus allows for early detection and rapid response to outbreaks. This includes the testing of both clinical samples from sick animals and the routine monitoring of apparently healthy herds [16]. When outbreaks occur, prompt and targeted intervention is crucial. This may include the isolation and treatment of sick animals, the depopulation of severely affected herds, and the implementation of additional biosecurity measures to prevent further spread.

Post-translational modification (PTM) represents a diverse and essential set of biochemical processes that occur after a protein has been synthesized from its corresponding gene [17]. These modifications involve the covalent attachment of functional groups or proteins, or the enzymatic alteration of specific amino acid residues within the protein backbone. PTMs encompass a wide range of activities, including phosphorylation, glycosylation, acetylation, methylation, ubiquitination, and many others [18]. They serve to fine-tune protein function, regulate protein activity, and facilitate protein interactions within the cell [19]. Accumulating evidence has shown that PTMs mediate infections of a variety of viruses. For example, severe acute respiratory syndrome coronavirus 2 (SARS-CoV-2) manipulates the glycosylation of the spike protein for viral entry and exploits the phosphorylation of the nucleocapsid protein to bind to viral RNA, thus contributing to viral replication [20]. Similarly, hepatitis B virus (HBV) also utilizes glycosylation, ubiquitination, acetylation, and phosphorylation to regulate self-replication in host cells via various mechanisms [21]. Recently, Johnson et al. depicted the global profiling of PTMs in human immunodeficiency virus (HIV)-infected cells and found that the Vpr protein suppresses histone H1 ubiquitination, impairing DNA repair and causing cell death [22].

## 2. Phosphorylation

PRRSV relies heavily on host cell machinery for its replication and pathogenesis. One crucial aspect of this interaction involves phosphorylation, which plays a pivotal role in virus–host dynamics. Phosphorylation is a process where a phosphate group is added to a protein, generally changing its functional properties. During PRRSV infection, phosphorylation can affect both viral proteins and host cell proteins, leading to altered viral replication, immune evasion, and pathogenesis.

### 2.1. On Viral Proteins

PRRSV proteins, particularly non-structural proteins (NSPs), undergo phosphorylation during the virus life cycle. This modification is believed to regulate their activities, such as RNA synthesis, protease activity, and antiviral immune responses. For instance, the phosphorylation of certain NSPs might enhance their ability to interact with host cell proteins, facilitating virus replication and assembly. It was reported that the phosphorylation of the N protein could regulate PRRSV replication and virulence in vivo and in vitro using the mutated virus by replacing the phosphorylation sites, with serine sites 105 and 120 primarily responsible for this regulation [23]. Similarly, a mutation of NS2 at phosphorylation site 918 could abrogate the production of recombinant viruses [24]. These studies suggest that the phosphorylation sites in PRRSV-encoded proteins have regulatory effects on PRRSV replication.

### 2.2. On Host Proteins

On the other hand, PRRSV also manipulates host cell phosphorylation pathways to create a favorable environment for its replication. The virus can alter the phosphorylation of specific host proteins, thereby altering cellular signaling cascades, immune responses, and apoptosis pathways. This modulation of host phosphorylation events allows PRRSV to evade the host immune system, promote cell survival, and enhance virus production. In 2014, Luo et al. determined the global levels of protein phosphorylation in pulmonary alveolar macrophages (PAMs) with PRRSV infection. They identified a total of 2125 phosphorylation sites in 966 proteins, which were further enriched in cellular organization, protein synthesis, and signal transduction [25]. While they did not elucidate the mechanisms underlying those proteins at that time, in recent years, many scientists have found a role for host protein phosphorylation in PRRSV infection. For example, PRRSV infection increases the phosphorylation of high-mobility group box 1 (HMGB1) at threonine-51 residue and enhance its interaction with ribosomal protein S3 (RPS3) to promote HMGB1 secretion, which in turn influences PRRSV replication [26]. In PRRSV-infected 3D4/21 cells, RNA-binding motif protein 39 (RBM39) expression is upregulated. RBM39 could alter c-Jun phosphorylation to inhibit the AP-1 pathway and promote PRRSV proliferation. This study reveals a complex mechanism regarding RBM39 in altering c-Jun phosphorylation, nucleocytoplasmic translocation, and regulating RBM39-viral RNA binding to promote PRRSV proliferation [27]. Interestingly, PRRSV could shut off host translation machinery by phosphorylating eukaryotic translation initiation factor 2A (eIF2α), which is related to NSP2 [28]. Vimentin, a component of intermediate filaments, has been implicated in viral infections. It was observed that PRRSV infection triggers post-entry vimentin reorganization, favoring viral replication. Specifically, vimentin is phosphorylated at serine residue 38 by calcium calmodulin-dependent protein kinase II gamma (CaMKIIγ), leading to the formation of cage-like vimentin structures that encapsulate the PRRSV replication complex [29].

**Figure 2:**
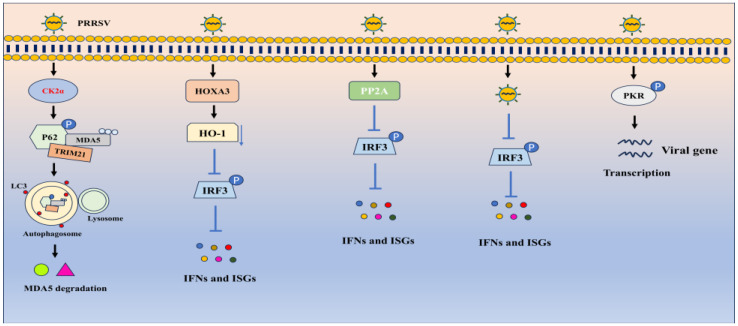
PRRSV utilizes phosphorylation to benefit self-replication. As shown, PRRSV could induce various host factors and pathways to block immune response, such as degrading MDA5 and inhibiting IRF3 phosphorylation, therefore contributing to enhanced viral replication.

Host innate immunity provides the first line of defense against PRRSV invasion [30]. While PRRSV enters the host, nuclear factor kappa B (NF-κB) signaling is activated upon IκB phosphorylation by the N protein, which subsequently contributes to interleukin (IL)-15 production and leads to impaired virus infection [31]. However, another study reported that the receptor of activated protein C kinase 1 (RACK1) promotes PRRSV replication by facilitating NF-κB signaling activation via increasing the expression and phosphorylation of TNF receptor-associated factor 2 (TRAF2) [32]. Yu et al. observed that PRRSV NSP12 could induce the phosphorylation of signal transducer and activator of transcription 1 (STAT1) at serine 727, which further causes high expression of IL-1β and IL-8 and ultimately attenuates PRRSV infection [33]. Moreover, the pro-inflammatory response could be induced by enhanced expression and phosphorylation of receptor-interacting protein 2 (RIP2) during PRRSV infection [34]. Nonetheless, PRRSV infection has evolved several strategies to antagonize host antiviral responses and benefit self-replication (Figure 2). For example, PRRSV utilizes homeobox A3 (HOXA3) to restrain interferon (IFN)-β and IFN-stimulated gene (ISG) expression in host cells. Further analysis showed that HOXA3 suppresses IFN regulating factor (IRF) 3 phosphorylation and nuclear translocation, thereby promoting PRRSV replication [35]. Similarly, PRRSV may enhance protein phosphatase 2A (PP2A) expression to dampen IRF3 phosphorylation and restrict IFN production, resulting in enhanced viral replication [36]. Surprisingly, Ren et al. found that PRRSV could inhibit IRF3 phosphorylation but not its nuclear translation to impede IFN-β expression [37]. Notably, PRRSV infection induces protein kinase R (PKR) phosphorylation, which in turn facilitates PRRSV replication [38]. On the other hand, PRRSV could enhance the degradation of the sensor melanoma differentiation-associated gene 5 (MDA5) via P62. Mechanistically, the interaction between P62 and MDA5 is enhanced by the phosphorylation of the P62 regulated by the kinase CK2α in PRRSV-infected cells [39]. These studies indicate that phosphorylation serves as a double-faced sword. Host cells exploit it to activate pathways for defense, whereas PRRSV utilizes it to counteract hose antiviral response.

## 3. Glycosylation

Glycosylation involves the attachment of carbohydrate moieties to proteins or lipids [40]. This process is crucial for the structural and functional integrity of numerous viral proteins, including those of PRRSV. PRRSV glycoproteins, particularly GP5, the most abundant glycoprotein, play a pivotal role in virus infectivity, antigenicity, and the ability to induce neutralizing antibodies [41]. These glycoproteins undergo N-linked glycosylation at specific sites, which are critical for their folding, stability, and immune recognition [42].

Studies have shown that the glycosylation of PRRSV GPs can significantly impact the viral ability to infect cells, evade the immune system, and replicate efficiently. For instance, the glycosylation status of GP5 can affect its binding to neutralizing antibodies, thereby influencing virus antigenic properties and immune evasion strategies [43]. It was also found that glycan addition at specific positions of GP2 and GP3 is crucial for virus production, and the glycosylation of GP2 and GP4 was key for efficient CD163 interaction, indicating the significance of glycosylation in the PRRSV life cycle [44]. However, according to Wei et al.’s findings, the N-linked glycosylation of GP2 is dispensable for viral viability, and neither of the glycosylation sites in GP3 play a crucial role in the generation of infectious viruses. Furthermore, their study indicated that mutating either single or double glycosylation sites in GP4 does not significantly hinder the recovery of infectious viruses [45]. Meanwhile, glycosylation in GP3 was found to affect the immune evasion of PRRSV [46]. Furthermore, glycosylation has been implicated in the modulation of PRRSV virulence. For example, Badaoui et al. analyzed the geographical and temporal distribution of PRRSV strains and further investigated the association between the glycosylation patterns in PRRSV sequences. As a result, they found that PRRSV with a glycosylation site at position N46 was observed to have a higher burden on pigs, and the infected pigs had a lower average daily gain compared with those infected with PRRSV lacking glycosylation at the N46 position site [47]. Additionally, Sui et al. compared the pathogenicity of two PRRSV stains, FZ06A and FZ16A, and observed that FZ06A caused higher mortality and virulence than FZ16A. Multiple genomic variations, including a unique N-glycosylation site mutation and an amino acid substitution in GP5 of FZ16A, were detected, highlighting the significance of glycosylation in virulence [48].

## 4. Ubiquitination

Ubiquitination is a critical PTM that involves the attachment of ubiquitin molecules to proteins, thereby regulating their function, localization, and lifespan within the cell [49]. Host cells also utilize ubiquitination as a defense mechanism by ubiquitinating and degrading viral proteins via proteasomal or lysosomal pathways.

### 4.1. On Viral Proteins

**Figure 3:**
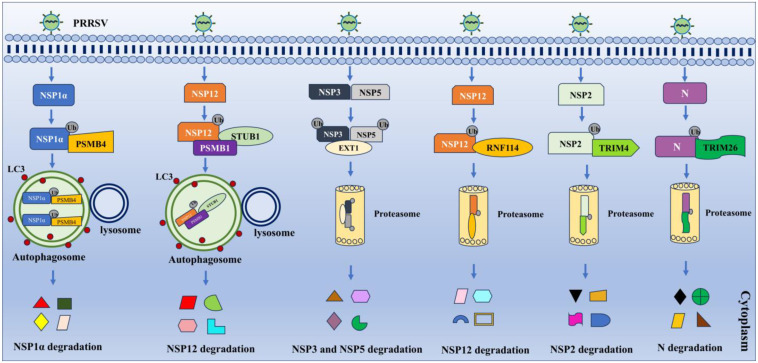
Host exploits ubiquitination to degrade viral proteins to suppress PRRSV replication. Viral proteins, including NSP1α, NSP12, NSP3, NSP5, NSP2, and N, could be recognized and ubiquitinated by different E3 ligase enzymes, finally delivered to autophagosome or proteasome for degradation, contributing to impaired viral replication.

RING finger protein 114 (RNF114) was shown to suppress the infection of PPRSV via the proteasomal degradation of NSP12, which is involved in K27-linked polyubiquitination [50]. Similarly, tripartite motif 4 (TRIM4) ubiquitinates and degrades NSP2 via the proteasomal pathway [51]. TRIM26 promotes the ubiquitination of K43 and K44 in N protein and results in proteasomal degradation, thus restricting PRRSV replication [52]. Proteasome subunit beta type 4 (PSMB4) targets NSP1α at K169 for K63-linked ubiquitination and lysosomal degradation to impair PRRSV replication [53]. Another member, PSMB1, recruits STIP1 homology U-box-containing protein 1 (STUB1) and the cargo receptor neighbor of BRCA1 gene 1 (NBR1) to ubiquitinate and degrade NSP12 via the autophagic lysosomal pathway [54]. Moreover, exostosin glycosyltransferase 1 (EXT1), associated with the biosynthesis of heparin sulfate, interacts with NSP3 and NSP5. EXT1 enhanced their K48-linked polyubiquitination to promote their proteasomal degradation [55]. These studies indicate that hosts exploit ubiquitination to targets and degrade viral proteins to impede PRRSV replication (Figure 3).

Additionally, ubiquitination could serve to stabilize proteins to aid in PRRSV infection. It is interesting to note that the host E3 ubiquitin ligase ankyrin repeat and SOCS box-containing 8 (ASB8) enhance the stability of NSP1α by promoting K63-linked ubiquitination, thus assisting in viral replication [56]. Surprisingly, TRIM28 stabilized GP4 by suppressing its ubiquitination to increase PRRSV infection [57]. The intricate interplay between PRRSV and ubiquitination underscores the complexity of virus–host interactions and highlights the potential of targeting this process for the development of novel antiviral therapies.

### 4.2. On Cellular Proteins

Accumulating studies have revealed that viruses hijack the host’s ubiquitination machinery to facilitate their own replication and evasion of the immune system. Specifically, they target key proteins within the host cell for ubiquitination, either to promote their degradation and suppress antiviral responses or to exploit their functions for viral replication [58]. Global ubiquitome analysis was performed in cells with PRRSV infection. A total of 4044 ubiquitination sites were identified on 1580 cellular proteins, with 983 sites significantly altered. Bioinformatic analysis showed that PRRSV manipulates the ubiquitination of key immune proteins like TNF receptor-associated factor 6 (TRAF6), Janus Kinase 1 (JAK1), and STAT1, suppressing the host’s immune response. For instance, PRRSV infection antagonizes the host’s innate immune response via recruiting TRIM21 to ubiquitinate MDA5 for degradation [39]. The PRRSV E protein induces autophagy and recruits P62 to degrade ubiquitinated DEAD-box helicase 10 (DDX10) to antagonize its antiviral effects [59]. Moreover, the E protein impedes the antiviral activity of cholesterol 25-hydroxylase (CH25H) by ubiquitination for proteasomal degradation [60]. Similarly, PRRSV also manipulates the immune regulator mucosa-associated lymphoid tissue lymphoma translocation protein 1 (MALT1) to degrade antiviral RNases, facilitating virus replication. However, PRRSV NSP6 mediates MALT1 degradation via the ubiquitination–proteasome pathway to suppress inflammatory responses, creating a balanced immune environment for viral settlement [61]. Additionally, the PRRSV N protein disrupts the interaction between TRIM25 and retinoic acid-inducible gene-I (RIG-I) through competitive binding with TRIM25. The N protein represses the expression of TRIM25 and hinders TRIM25-induced RIG-I ubiquitination, ultimately suppressing the production of IFN-β [62].

## 5. SUMOylation

SUMOylation, involving the attachment of small ubiquitin-like modifier (SUMO) proteins to other proteins, plays a crucial role in regulating various cellular processes [63]. These processes include protein–protein interactions, protein localization, and transcriptional activity. In PRRSV infection, SUMOylation has emerged as an important factor that can influence both the viral lifecycle and the host response to the infection. PRRSV has been found to manipulate the host SUMOylation machinery to facilitate its own replication and evasion of the immune system.

### 5.1. On Viral Proteins

Wang et al. found that multiple PRRSV proteins could undergo SUMOylation. They confirmed interactions between PRRSV proteins (NSP1β, NSP4, NSP9, NSP10, and N) and the SUMO E2 enzyme Ubc9. Ubc9 co-localized with these proteins in specific cellular compartments. They demonstrated that the N protein can be SUMOylated by SUMO1 or SUMO2/3. Moreover, altered Ubc9 expression affected PRRSV replication, suggesting its involvement in this process [64].

### 5.2. On Cellular Proteins

For instance, Shi et al. found that PRRSV NSP1α facilitates viral escape from the innate immune system by modulating nuclear to cytoplasmic translocation and the distribution ratio of TRAF-interacting protein (TRAIP) to promote virus proliferation. Mechanistically, TRAIP interacts with PRRSV NSP1α via its K205 site, while NSP1α decreases SUMOylation and K48 ubiquitination independent of TRAIP’s interaction with the K205 site. Modulation of the dual modification of TRAIP by PRRSV NSP1α results in over-enrichment of TRAIP in the cytoplasm. The enrichment of NSP1α-induced cytoplasmic TRAIP, in turn, leads to excessive K48 ubiquitination and the degradation of serine/threonine-protein kinase (TBK1), thereby antagonizing TBK1-IRF3-IFN signaling [65].

## 6. Acetylation

Acetylation involves the addition of acetyl groups to proteins, altering their functions and interactions within the cell [66]. On the host side, the acetylation of cellular proteins can modulate the signaling pathways against PRRSV infection. For example, the acetylation of certain transcription factors may enhance or suppress the expression of genes involved in antiviral defense, thereby shaping the host overall response to the virus.

### 6.1. On Viral Proteins

On the viral side, acetylation can modify the properties of viral proteins, influencing their stability, localization, and ability to interact with host cell components [67]. For instance, Li et al. revealed that endoplasmic reticulum-resident N-acetyltransferase Nat9 acts as a key host restriction factor for PRRSV infection. Nat9 hinders PRRSV proliferation through its acetyltransferase activity. Specifically, GP5 interacts with and is acetylated by Nat9. This acetylation generates a degradation signal for GP5, promoting its K27-linked ubiquitination and degradation, thereby reducing virion assembly. However, they further identified ETV5 and SP1 as crucial transcription factors binding to the Nat9 promoter region. PRRSV proteins NSP1β, NSP4, NSP9, and N interfere significantly with ETV5 and SP1 expression, regulating Nat9 transcription and inhibiting its expression. This suggests that PRRSV inhibits Nat9 to reduce N-terminal acetylation of GP5, favoring virion assembly [68].

### 6.2. On Cellular Proteins

Fang et al. performed a global acetylome analysis to study alterations of acetylation in PRRSV-infected cells. They identified 3731 lysine acetylation sites on 1421 cellular proteins, involving various biological processes like immune response and energy metabolism. They found that a large number of immune factors, including CREB-binding protein (CBP) and ISG15, as well as inflammatory regulators, including heat shock protein 70 (HSP70) and cathepsin B, are altered with different acetylation levels [69]. This contributes to understanding PRRSV pathogenesis and aids in identifying new targets for anti-PPRSV therapeutics.

## 7. Palmitoylation

Palmitoylation involves the covalent attachment of palmitate, a fatty acid, to specific cysteine residues of proteins, and it has been shown to affect various aspects of viral infections and virus–host interactions [70]. Palmitoylation can influence the membrane association and subcellular localization of viral proteins. Many viral proteins, especially those involved in membrane fusion, budding, or viral assembly, require precise membrane targeting to perform their functions. Palmitoylation enhances the hydrophobicity of these proteins, facilitating their interaction with cellular membranes and potentially altering their localization within the infected cell [71,72]. In addition, palmitoylation may affect enzymatic activity, protein–protein interactions, or even the stability of proteins [73]. These alterations can, in turn, impact the efficiency of virus replication, virus assembly, or the ability of the virus to evade the host immune response.

### 7.1. On Viral Proteins

It was shown that the cysteines in GP5 and M of PRRSV-1 and -2 undergo palmitoylation, which is essential for virus growth. Notably, the substitution of cysteines reduces palmitoylation. Additionally, the palmitoylation of GP5 influences PRRSV budding by the removal of palmitoylation sites from GP5. However, a lack of palmitoylation in M and GP5 does not cause deficiency in viral entry or trafficking to the budding sites [74].

### 7.2. On Cellular Proteins

Zhang et al. found that enhancing IFN-induced transmembrane protein 3 (IFITM3) reduces PRRSV replication, while suppressing it boosts virus growth. IFITM3 undergoes S-palmitoylation and ubiquitination modifications crucial to its antiviral activity. PRRSV enters endosomes and lysosomes early in infection, and IFITM3 redirects it to cholesterol-rich vesicles, limiting membrane fusion. IFITM3 also reduces PRRSV infectivity and spread between cells [75].

## 8. Lactylation

Lactic acid production, or more specifically, the role of lactate dehydrogenase (LDH) in the context of PRRSV, plays a significant part in the viral replication cycle and the host metabolic response to infection. During PRRSV infection, the virus hijacks the host machinery to replicate its genetic material and produce new virus particles. This process is energy-intensive and often leads to the metabolic reprogramming of the infected cell. One such change involves an increase in glycolytic activity, which is the breakdown of glucose into ATP and lactic acid. LDH, a key enzyme in this pathway, converts pyruvate to lactate, generating energy for the cell and the virus [76].

Studies by Zhang et al. have indicated that PRRSV infection induces the upregulation of lactate and LDH. This upregulation is essential for efficient virus replication as it provides the necessary energy and biosynthetic precursors. Additionally, the accumulation of lactic acid in the extracellular environment can also affect the immune response by targeting MAVS that may favor virus survival [77]. Furthermore, lactate has recently been identified as a substrate for the formation of lactylation [78]. Most recently, Pang et al. observed and discovered that lactate boosts PRRSV growth and that PRRSV infection raises lactylation levels. They identified genes affected by lactylation in infected cells, finding that PRRSV-induced lactylation activates HSPA6 gene expression. Follow-up tests showed that HSPA6 aids PRRSV growth by reducing IFN-β production. This occurs because HSPA6 disrupts the interaction between TRAF3 and IKKε, limiting IFN-β creation [79].

## 9. Development of PTMs as Novel Strategies and Therapeutic Targets

The development of PTMs as novel strategies and therapeutic targets against viral infections marks a pivotal shift in our approach to combating viral pathogens. In the realm of virology, these modifications have emerged as critical determinants of viral replication, assembly, and immune evasion. One compelling example is the phosphorylation of viral proteins, a process that can activate or inhibit their function, thereby influencing viral life cycles. For instance, the phosphorylation of the Ebola virus VP30 protein by host kinases enhances its ability to interact with the viral RNA-dependent RNA polymerase, promoting efficient viral replication [80]. By designing small-molecule inhibitors that target this phosphorylation event, viral replication could be disrupted in vitro. Another example is the ubiquitination of viral proteins, a modification that can target proteins for degradation or alter their subcellular localization. In the case of the HIV-1 virus, the SUMOylation and ubiquitination of Gag p6 are essential for virus assembly and budding from infected cells [81]. Developing compounds that inhibit these processes could impair HIV-1 particle formation, thus blocking viral spread. More recently, the inhibitor ST1326, targeting succinylation, reduces the replication of SARS-CoV-2, underscoring the significance of succinylation in SARS-CoV-2 infection [82].

These PTMs involve the targeted alteration of specific proteins that play pivotal roles in the viral replication cycle or modulate host response pathways, with the ultimate goal of disrupting disease progression and fortifying porcine inherent resistance against the virus. The SUMOylation of the PRRSV N protein, for instance, has been shown to play a pivotal role in viral replication and assembly [66]. By developing compounds that specifically inhibit this, PRRSV replication might be impaired in vitro. Furthermore, glycosylation has also been implicated in PRRSV infections. The glycosylation of PRRSV envelope proteins, such as GP5, is essential for virus entry into host cells and immune evasion [41]. Inhibiting the enzymes responsible for this glycosylation could reduce viral infectivity and enhance the recognition of viral particles by the host immune system. Additionally, protein modification strategies could also be employed to enhance the efficacy of existing vaccines, making them more potent against the virus. For example, both serine 105 and 120 were phosphorylation sites that contributed to reduced PRRSV titers and virulence, indicating an important role of these sites for the PRRSV life cycle [23]. In view of this, future vaccines could consider designing vaccines with phosphorylation site mutations.

These examples illustrate the profound impact that PTMs can have on viral biology and the potential for targeting these modifications as a therapeutic strategy. As our understanding of PTMs in viral infections continues to deepen, so too does our ability to harness this knowledge for the development of novel antivirals that are more specific, effective, and less prone to resistance. This represents a promising new frontier in the fight against viral diseases, with the potential to revolutionize antiviral therapy and improve global public health.

## 10. Conclusions

PTMs profoundly impact the biology and pathogenicity of PRRSV. These modifications, occurring after protein synthesis, introduce chemical changes that alter the structures, functions, and interactions of both host proteins (Table 1) and even viral proteins (Table 2). PTMs such as succinylation and crotonylation hold promise in further elucidating PRRSV’s complex biology. Succinylation, involving the addition of succinate groups to proteins, has been implicated in regulating metabolic enzymes and mitochondrial function in other systems [83]. Given PRRSV’s reliance on the host cell’s metabolism, exploring the role of succinylation in shaping the viral life cycle could offer novel insights. Crotonylation, a more recently identified PTM involving the attachment of crotonyl groups, has been associated with transcriptional regulation and epigenetic modifications [84]. Investigating whether and how PRRSV proteins undergo crotonylation could reveal new layers of virus–host interactions and potential epigenetic reprogramming strategies employed by the virus.

Notably, Although PRRSV does not directly infect humans, it may indirectly affect human health through the following ways. Firstly, infected pigs may carry the virus and contaminate pork products. Although the virus can be inactivated during high-temperature cooking, raw or inadequately cooked pork products may pose a potential health risk. Secondly, PRRSV causes significant economic losses to the pork industry, which may lead to a shortage of pork supply and price increases. Pork is one of the important protein sources for humans, and price increases may affect consumers’ food expenditure and quality of life. Pig farm workers may become carriers of the virus after contact with infected pigs or contaminants. However, since PRRSV is not directly infectious to human cells, the workers will not become a source of virus transmission. The virus may also spread within or near the pig farm through environmental media such as air, water, and soil. Although PRRSV has no direct impact on human health, it may indirectly affect human health through food safety issues, economic impacts, and animal welfare and ethical concerns.

In conclusion, the study of PTMs in PRRSV continues to unveil the intricate ways in which the virus manipulates host cell machinery for its survival and replication. Future research into less explored modifications like succinylation and crotonylation holds the potential to further expand our understanding of PRRSV pathogenesis and pave the way for innovative therapeutic approaches.

## Figures and Tables

**Table 1:** Effects of host protein modifications on PRRSV.

Protein Modifications	Host Proteins	Effects on Viruses
Phosphorylation	HMGB1	It enhances interaction with RPS3 to promote HMGB1 secretion to affect PRRSV [26].
	c-Jun	RBM39 alters c-JUN phosphorylation to suppress the AP-1 pathway and promotes PRRSV proliferation [27].
	eIF2a	It shuts off host translation machinery [28].
	Vimentin	It contributes to the formation of cage-like vimentin structures that encapsulate the PRRSV replication complex [29].
	IκB	It activates NF-κB and promotes IL-15 production, impairing PRRSV infection [31].
	TRAF2	It facilitates NF-κB signaling to promote PRRSV infection [32].
	STAT1	It elevates IL-1β and IL-8 production and attenuates PRRSV infection [33].
	RIP2	It induces pro-inflammatory effects and impairs PRRSV infection [34].
	IRF3	HOXA3 restrains IRF3 phosphorylation and promotes PRRSV infection [35].
	PKR	PRRSV induces PKR phosphorylation and, in turn, facilitates PRRSV replication [38].
	P62	It enhances interaction with MDA5 and promotes the degradation of MDA5, attenuating innate immunity and boosting PRRSV infection [39].
Ubiquitination	MDA5	TRIM21 ubiquitinates MDA5 for degradation, impairing innate immunity and aiding in viral replication [39].
	CH25H	E promotes CH25H ubiquitination and degradation, impeding innate response and promoting viral replication [60].
	MALT1	NSP6 mediates MALT ubiquitination, preventing MALT from degrading RNases for viral settlement [61].
	RIG-I	N disrupts the interaction between TRIM25 and RIG-I and suppresses the TRIM25-induced ubiquitination of RIG-I, restraining innate signaling and promoting viral growth [62].
SUMOylation	TRAIP	NSP1α-induced SUMOylation of TRAIP causes the degradation of TBK1, antagonizing innate signaling and favoring viral replication [65].
Acetylation	ISG15	Unknown [69].
	HSP70	Unknown [69].
Palmitoylation	IFITM3	It reduces PRRSV infectivity and spread, limiting membrane fusion [75].

**Table 2:** PRRSV proteins modified with various PTMs.

Protein Modifications	Viral Proteins	Effects on PRRSV
Phosphorylation	N	N phosphorylation regulates PRRSV replication and virulence in vivo and in vitro [23].
	NSP2	NSP2 phosphorylation modulates the production of recombinant PRRSV [24].
Glycosylation	GP5	GP5 glycosylation affects binding with neutralizing antibodies and influences the virus’s antigenic properties and immune evasion strategies [41].
	GP2	GP2 glycosylation makes CD163 interaction more efficient and is crucial for virus production [44].
	GP3	It is crucial for virus production [44].
	GP4	It causes efficient CD163 interaction [44].
Ubiquitination	NSP12	RNF114 ubiquitinates NSP12 for degradation, attenuating PRRSV replication [50].
	NSP2	TRIM4 ubiquitinates NSP2 for degradation, impairing PRRSV replication [51].
	N	TRIM26 promotes the ubiquitination of N and restricts PRRSV replication [52].
	NSP1α	PSMB4 targets NSP1α for ubiquitination and degradation, impairing PRRSV replication [53].
	NSP3	EXT1 enhances the ubiquitination of NSP3 and suppresses PRRSV replication [55].
	NSP5	EXT1 enhances NSP5 ubiquitination to attenuate PRRSV replication [55].
SUMOylation	NSP1β	Unknown [64].
	NSP4	Unknown [64].
	NSP9	Unknown [64].
	NSP10	Unknown [64].
Acetylation	GP5	Nat9 promotes the acetylation of GP5 and hinders PRRSV infection [68].
Palmitoylation	GP5	GP5 palmitoylation affects PRRSV release and growth [74].
	M	M palmitoylation affects PRRSV release and growth [74].

## References

[1] Lunney J.K., Fang Y., Ladinig A., Chen N., Li Y., Rowland B., Renukaradhya G.J. (2016). Porcine Reproductive and Respiratory Syndrome Virus (PRRSV): Pathogenesis and Interaction with the Immune System. Annu. Rev. Anim. Biosci..

[2] Yi H., Ye R., Xie E., Lu L., Wang Q., Wang S., Sun Y., Tian T., Qiu Y., Wu Q. (2024). ZNF283, a Krüppel-Associated Box Zinc Finger Protein, Inhibits RNA Synthesis of Porcine Reproductive and Respiratory Syndrome Virus by Interacting with Nsp9 and Nsp10. Vet. Res..

[3] Zhang Z., Wen X., Dong J., Ge X., Zhou L., Yang H., Guo X. (2017). Epitope Mapping and Characterization of a Novel Nsp10-Specific Monoclonal Antibody That Differentiates Genotype 2 PRRSV from Genotype 1 PRRSV. Virol. J..

[4] An T.-Q., Li J.-N., Su C.-M., Yoo D. (2020). Molecular and Cellular Mechanisms for PRRSV Pathogenesis and Host Response to Infection. Virus Res..

[5] Zheng Y., Li G., Luo Q., Sha H., Zhang H., Wang R., Kong W., Liao J., Zhao M. (2024). Research Progress on the N Protein of Porcine Reproductive and Respiratory Syndrome Virus. Front. Microbiol..

[6] Chen X., Pan J., Huang L., Zhao M. (2023). Research Progress on the E Protein of Porcine Reproductive and Respiratory Syndrome Virus. Front. Microbiol..

[7] Sun Y., Chen Y., Cai Y., Li Q., Xie J., Liang G., Gao Q., Yu Z., Lu G., Huang L. (2020). Insights into the Evolutionary History and Epidemiological Characteristics of the Emerging Lineage 1 Porcine Reproductive and Respiratory Syndrome Viruses in China. Transbound. Emerg. Dis..

[8] Carlsson U., Wallgren P., Renström L.H.M., Lindberg A., Eriksson H., Thorén P., Eliasson-Selling L., Lundeheim N., Nörregard E., Thörn C. (2009). Emergence of Porcine Reproductive and Respiratory Syndrome in Sweden: Detection, Response and Eradication. Transbound. Emerg. Dis..

[9] Han J., Zhou L., Ge X., Guo X., Yang H. (2017). Pathogenesis and Control of the Chinese Highly Pathogenic Porcine Reproductive and Respiratory Syndrome Virus. Vet. Microbiol..

[10] Pileri E., Mateu E. (2016). Review on the Transmission Porcine Reproductive and Respiratory Syndrome Virus between Pigs and Farms and Impact on Vaccination. Vet. Res..

[11] Sykes A.L., Silva G.S., Holtkamp D.J., Mauch B.W., Osemeke O., Linhares D.C.L., Machado G. (2022). Interpretable Machine Learning Applied to On-Farm Biosecurity and Porcine Reproductive and Respiratory Syndrome Virus. Transbound. Emerg. Dis..

[12] Rahe M.C., Murtaugh M.P. (2017). Mechanisms of Adaptive Immunity to Porcine Reproductive and Respiratory Syndrome Virus. Viruses.

[13] Guo Z., Chen X.-X., Li R., Qiao S., Zhang G. (2018). The Prevalent Status and Genetic Diversity of Porcine Reproductive and Respiratory Syndrome Virus in China: A Molecular Epidemiological Perspective. Virol. J..

[14] Yu F., Liu L., Tian X., Chen L., Huang X., Sun Y., Yan Y., Tian Z., Cai X., Liu D. (2022). Genomic Analysis of Porcine Reproductive and Respiratory Syndrome Virus 1 Revealed Extensive Recombination and Potential Introduction Events in China. Vet. Sci..

[15] Lambert M.-È., Audet P., Delisle B., Arsenault J., D’Allaire S. (2019). Porcine Reproductive and Respiratory Syndrome Virus: Web-Based Interactive Tools to Support Surveillance and Control Initiatives. Porc. Health Manag..

[16] Baliellas J., Novell E., Enric-Tarancón V., Vilalta C., Fraile L. (2021). Porcine Reproductive and Respiratory Syndrome Surveillance in Breeding Herds and Nurseries Using Tongue Tips from Dead Animals. Vet. Sci..

[17] Campbell S.L., Philips M.R. (2021). Post-Translational Modification of RAS Proteins. Curr. Opin. Struct. Biol..

[18] Lin H., Caroll K.S. (2018). Introduction: Posttranslational Protein Modification. Chem. Rev..

[19] Liu J., Qian C., Cao X. (2016). Post-Translational Modification Control of Innate Immunity. Immunity.

[20] Cheng N., Liu M., Li W., Sun B., Liu D., Wang G., Shi J., Li L. (2022). Protein Post-Translational Modification in SARS-CoV-2 and Host Interaction. Front. Immunol..

[21] Yang F. (2018). Post-Translational Modification Control of HBV Biological Processes. Front. Microbiol..

[22] Johnson J.R., Crosby D.C., Hultquist J.F., Kurland A.P., Adhikary P., Li D., Marlett J., Swann J., Hüttenhain R., Verschueren E. (2022). Global Post-Translational Modification Profiling of HIV-1-Infected Cells Reveals Mechanisms of Host Cellular Pathway Remodeling. Cell Rep..

[23] Chen Y., Yu Z., Yi H., Wei Y., Han X., Li Q., Ji C., Huang J., Deng Q., Liu Y. (2019). The Phosphorylation of the N Protein Could Affect PRRSV Virulence In Vivo. Vet. Microbiol..

[24] Shang P., Yuan F., Misra S., Li Y., Fang Y. (2020). Hyper-Phosphorylation of Nsp2-Related Proteins of Porcine Reproductive and Respiratory Syndrome Virus. Virology.

[25] Luo R., Fang L., Jin H., Wang D., An K., Xu N., Chen H., Xiao S. (2014). Label-Free Quantitative Phosphoproteomic Analysis Reveals Differentially Regulated Proteins and Pathway in PRRSV-Infected Pulmonary Alveolar Macrophages. J. Proteome Res..

[26] Wang R., Zhang J., Fu Y., Jia L., Zhang Y., Bai L., Wang W., Cheng D., Liu E. (2022). PRRSV Induces HMGB1 Phosphorylation at Threonine-51 Residue to Enhance Its Secretion. Viruses.

[27] Song Y., Guo Y., Li X., Sun R., Zhu M., Shi J., Tan Z., Zhang L., Huang J. (2021). RBM39 Alters Phosphorylation of C-Jun and Binds to Viral RNA to Promote PRRSV Proliferation. Front. Immunol..

[28] Li Y., Fang L., Zhou Y., Tao R., Wang D., Xiao S. (2018). Porcine Reproductive and Respiratory Syndrome Virus Infection Induces Both EIF2α Phosphorylation-Dependent and -Independent Host Translation Shutoff. J. Virol..

[29] Zheng X.-X., Li R., Qiao S., Chen X.-X., Zhang L., Lu Q., Xing G., Zhou E.-M., Zhang G. (2021). Vimentin Rearrangement by Phosphorylation Is Beneficial for Porcine Reproductive and Respiratory Syndrome Virus Replication In Vitro. Vet. Microbiol..

[30] Chen X., Li Z., Wang S., Tong G., Chen K., Zhao Y. (2022). Proteomic Analysis Reveals Zinc-Finger CCHC-Type Containing Protein 3 as a Factor Inhibiting Virus Infection by Promoting Innate Signaling. Virus Res..

[31] Fu Y., Quan R., Zhang H., Hou J., Tang J., Feng W. (2012). Porcine Reproductive and Respiratory Syndrome Virus Induces Interleukin-15 through the NF-ΚB Signaling Pathway. J. Virol..

[32] Liu X., Bi J., Zhao Q., Li M., Zuo Q., Wang X., Lan R., Li X., Yang G., Liu J. (2019). Overexpression of RACK1 Enhanced the Replication of Porcine Reproductive and Respiratory Syndrome Virus in Marc-145 Cells and Promoted the NF-ΚB Activation via Upregulating the Expression and Phosphorylation of TRAF2. Gene.

[33] Yu Y., Wang R., Nan Y., Zhang L., Zhang Y. (2013). Induction of STAT1 Phosphorylation at Serine 727 and Expression of Proinflammatory Cytokines by Porcine Reproductive and Respiratory Syndrome Virus. PLoS ONE.

[34] Jing H., Fang L., Wang D., Ding Z., Luo R., Chen H., Xiao S. (2014). Porcine Reproductive and Respiratory Syndrome Virus Infection Activates NOD2–RIP2 Signal Pathway in MARC-145 Cells. Virology.

[35] Feng Y., Guo X., Tian H., He Y., Li Y., Jiang X., Zheng H., Xiao S. (2022). Induction of HOXA3 by Porcine Reproductive and Respiratory Syndrome Virus Inhibits Type I Interferon Response through Negative Regulation of HO-1 Transcription. J. Virol..

[36] Xu J., Zhang L., Xu Y., Zhang H., Gao J., Wang Q., Tian Z., Xuan L., Chen H., Wang Y. (2019). PP2A Facilitates Porcine Reproductive and Respiratory Syndrome Virus Replication by Deactivating Irf3 and Limiting Type I Interferon Production. Viruses.

[37] Ren Y., Khan F.A., Pandupuspitasari N.S., Li S., Hao X., Chen X., Xiong J., Yang L., Fan M., Zhang S. (2016). Highly Pathogenic Porcine Reproductive and Respiratory Syndrome Virus Modulates Interferon-β Expression Mainly Through Attenuating Interferon-Regulatory Factor 3 Phosphorylation. DNA Cell Biol..

[38] Wang X., Zhang H., Abel A.M., Nelson E. (2016). Protein Kinase R (PKR) Plays a pro-Viral Role in Porcine Reproductive and Respiratory Syndrome Virus (PRRSV) Replication by Modulating Viral Gene Transcription. Arch. Virol..

[39] Sun R., Guo Y., Zhang L., Zhang H., Yin B., Li X., Li C., Yang L., Zhang L., Li Z. (2024). PRRSV Degrades MDA5 via Dual Autophagy Receptors P62 and CCT2 to Evade Antiviral Innate Immunity. Virol. Sin..

[40] Eichler J. (2019). Protein Glycosylation. Curr. Biol..

[41] Wei Z., Lin T., Sun L., Li Y., Wang X., Gao F., Liu R., Chen C., Tong G., Yuan S. (2012). N-Linked Glycosylation of GP5 of Porcine Reproductive and Respiratory Syndrome Virus Is Critically Important for Virus Replication In Vivo. J. Virol..

[42] Paploski I.A.D., Makau D.N., Pamornchainavakul N., Baker J.P., Schroeder D., Rovira A., VanderWaal K. (2022). Potential Novel N-Glycosylation Patterns Associated with the Emergence of New Genetic Variants of PRRSV-2 in the U.S. Vaccines.

[43] Ansari I.H., Kwon B., Osorio F.A., Pattnaik A.K. (2006). Influence of N-Linked Glycosylation of Porcine Reproductive and Respiratory Syndrome Virus GP5 on Virus Infectivity, Antigenicity, and Ability to Induce Neutralizing Antibodies. J. Virol..

[44] Das P.B., Vu H.L.X., Dinh P.X., Cooney J.L., Kwon B., Osorio F.A., Pattnaik A.K. (2011). Glycosylation of Minor Envelope Glycoproteins of Porcine Reproductive and Respiratory Syndrome Virus in Infectious Virus Recovery, Receptor Interaction, and Immune Response. Virology.

[45] Wei Z., Tian D., Sun L., Lin T., Gao F., Liu R., Tong G., Yuan S. (2012). Influence of N-Linked Glycosylation of Minor Proteins of Porcine Reproductive and Respiratory Syndrome Virus on Infectious Virus Recovery and Receptor Interaction. Virology.

[46] Vu H.L.X., Kwon B., Yoon K.-J., Laegreid W.W., Pattnaik A.K., Osorio F.A. (2011). Immune Evasion of Porcine Reproductive and Respiratory Syndrome Virus through Glycan Shielding Involves Both Glycoprotein 5 as Well as Glycoprotein 3. J. Virol..

[47] Badaoui B., Grande R., Calza S., Cecere M., Luini M., Stella A., Botti S. (2013). Impact of Genetic Variation and Geographic Distribution of Porcine Reproductive and Respiratory Syndrome Virus on Infectivity and Pig Growth. BMC Vet. Res..

[48] Sui X., Xin T., Guo X., Jia H., Li M., Gao X., Wu J., Jiang Y., Willems L., Zhu H. (2018). Genomic Characterization and Pathogenic Study of Two Porcine Reproductive and Respiratory Syndrome Viruses with Different Virulence in Fujian, China. J. Vet. Sci..

[49] Popovic D., Vucic D., Dikic I. (2014). Ubiquitination in Disease Pathogenesis and Treatment. Nat. Med..

[50] Bai Y., Li L., Shan T., Zhang Y., Chen X., Gao F., Jiang Y., Zhou Y., Li G., Yu L. (2020). Proteasomal Degradation of Nonstructural Protein 12 by RNF114 Suppresses Porcine Reproductive and Respiratory Syndrome Virus Replication. Vet. Microbiol..

[51] Zhao M., Sha H., Zhang H., Wang R. (2022). TRIM4-Mediated Ubiquitination of NSP2 Restricts Porcine Reproductive and Respiratory Syndrome Virus Proliferation. BMC Vet. Res..

[52] Zhao P., Jing H., Dong W., Duan E., Ke W., Tao R., Li Y., Cao S., Wang H., Zhang Y. (2022). TRIM26-Mediated Degradation of Nucleocapsid Protein Limits Porcine Reproductive and Respiratory Syndrome Virus-2 Infection. Virus Res..

[53] Yi H., Wang Q., Lu L., Ye R., Xie E., Yu Z., Sun Y., Chen Y., Cai M., Qiu Y. (2023). PSMB4 Degrades the Porcine Reproductive and Respiratory Syndrome Virus Nsp1α Protein via the Autolysosome Pathway and Induces the Production of Type I Interferon. J. Virol..

[54] Li L., Bai Y., Zhou Y., Jiang Y., Tong W., Li G., Zheng H., Gao F., Tong G. (2023). PSMB1 Inhibits the Replication of Porcine Reproductive and Respiratory Syndrome Virus by Recruiting NBR1 To Degrade Nonstructural Protein 12 by Autophagy. J. Virol..

[55] Zhang X., Dong W., Wang X., Zhu Z., He S., Zhang H., Chen Y., Liu X., Guo C. (2022). Exostosin Glycosyltransferase 1 Reduces Porcine Reproductive and Respiratory Syndrome Virus Infection through Proteasomal Degradation of Nsp3 and Nsp5. J. Biol. Chem..

[56] Li R., Chen C., He J., Zhang L., Zhang L., Guo Y., Zhang W., Tan K., Huang J. (2019). E3 Ligase ASB8 Promotes Porcine Reproductive and Respiratory Syndrome Virus Proliferation by Stabilizing the Viral Nsp1α Protein and Degrading Host IKKβ Kinase. Virology.

[57] Cui Z., Zhou L., Zhao S., Li W., Li J., Chen J., Zhang Y., Xia P. (2023). The Host E3-Ubiquitin Ligase TRIM28 Impedes Viral Protein GP4 Ubiquitination and Promotes PRRSV Replication. Int. J. Mol. Sci..

[58] Song K., Li S. (2021). The Role of Ubiquitination in NF-ΚB Signaling during Virus Infection. Viruses.

[59] Li J., Zhou Y., Zhao W., Liu J., Ullah R., Fang P., Fang L., Xiao S. (2023). Porcine Reproductive and Respiratory Syndrome Virus Degrades DDX10 via SQSTM1/P62-Dependent Selective Autophagy to Antagonize Its Antiviral Activity. Autophagy.

[60] Ke W., Fang L., Tao R., Li Y., Jing H., Wang D., Xiao S. (2019). Porcine Reproductive and Respiratory Syndrome Virus E Protein Degrades Porcine Cholesterol 25-Hydroxylase via the Ubiquitin-Proteasome Pathway. J. Virol..

[61] Gu H., Zheng S., Han G., Yang H., Deng Z., Liu Z., He F. (2022). Porcine Reproductive and Respiratory Syndrome Virus Adapts Antiviral Innate Immunity via Manipulating MALT1. mBio.

[62] Zhao K., Li L.-W., Jiang Y.-F., Gao F., Zhang Y.-J., Zhao W.-Y., Li G.-X., Yu L.-X., Zhou Y.-J., Tong G.-Z. (2019). Nucleocapsid Protein of Porcine Reproductive and Respiratory Syndrome Virus Antagonizes the Antiviral Activity of TRIM25 by Interfering with TRIM25-Mediated RIG-I Ubiquitination. Vet. Microbiol..

[63] Henley J.M., Carmichael R.E., Wilkinson K.A. (2018). Extranuclear SUMOylation in Neurons. Trends Neurosci..

[64] Wang C., Zeng N., Liu S., Miao Q., Zhou L., Ge X., Han J., Guo X., Yang H. (2017). Interaction of Porcine Reproductive and Respiratory Syndrome Virus Proteins with SUMO-Conjugating Enzyme Reveals the SUMOylation of Nucleocapsid Protein. PLoS ONE.

[65] Shi P., Su Y., Li R., Zhang L., Chen C., Zhang L., Faaberg K., Huang J. (2018). Dual Regulation of Host TRAIP Post-Translation and Nuclear/Plasma Distribution by Porcine Reproductive and Respiratory Syndrome Virus Non-Structural Protein 1α Promotes Viral Proliferation. Front. Immunol..

[66] Baeza J., Smallegan M.J., Denu J.M. (2016). Mechanisms and Dynamics of Protein Acetylation in Mitochondria. Trends Biochem. Sci..

[67] Serman T., Chiang C., Liu G., Sayyad Z., Pandey S., Volcic M., Lee H., Muppala S., Acharya D., Goins C. (2023). Acetylation of the NS3 Helicase by KAT5γ Is Essential for Flavivirus Replication. Cell Host Microbe.

[68] Li X., Sun R., Guo Y., Zhang H., Xie R., Fu X., Zhang L., Zhang L., Li Z., Huang J. (2023). N-Acetyltransferase 9 Inhibits Porcine Reproductive and Respiratory Syndrome Virus Proliferation by N-Terminal Acetylation of the Structural Protein GP5. Microbiol. Spectr..

[69] Fang J., Qiao S., Wang K., Li R., Wang L., Li H., Zhang G. (2021). Quantitative Proteomic Analysis of Global Protein Acetylation in PRRSV-Infected Pulmonary Alveolar Macrophages. Proteomics.

[70] Veit M. (2012). Palmitoylation of Virus Proteins. Biol. Cell.

[71] Ma X., Xia Q., Liu K., Wu Z., Li C., Xiao C., Dong N., Hameed M., Anwar M.N., Li Z. (2023). Palmitoylation at Residue C221 of Japanese Encephalitis Virus NS2A Protein Contributes to Viral Replication Efficiency and Virulence. J. Virol..

[72] Veit M., Serebryakova M.V., Kordyukova L. (2013). V Palmitoylation of Influenza Virus Proteins. Biochem. Soc. Trans..

[73] Hao S., Zheng X., Zhu Y., Yao Y., Li S., Xu Y., Feng W.-H. (2023). African Swine Fever Virus QP383R Dampens Type I Interferon Production by Promoting CGAS Palmitoylation. Front. Immunol..

[74] Zhang M., Han X., Osterrieder K., Veit M. (2021). Palmitoylation of the Envelope Membrane Proteins GP5 and M of Porcine Reproductive and Respiratory Syndrome Virus Is Essential for Virus Growth. PLoS Pathog..

[75] Zhang A., Duan H., Zhao H., Liao H., Du Y., Li L., Jiang D., Wan B., Wu Y., Ji P. (2020). Interferon-Induced Transmembrane Protein 3 Is a Virus-Associated Protein Which Suppresses Porcine Reproductive and Respiratory Syndrome Virus Replication by Blocking Viral Membrane Fusion. J. Virol..

[76] Flores A., Schell J., Krall A.S., Jelinek D., Miranda M., Grigorian M., Braas D., White A.C., Zhou J.L., Graham N.A. (2017). Lactate Dehydrogenase Activity Drives Hair Follicle Stem Cell Activation. Nat. Cell Biol..

[77] Zhang L., Liu X., Mao J., Sun Y., Gao Y., Bai J., Jiang P. (2023). Porcine Reproductive and Respiratory Syndrome Virus-Mediated Lactate Facilitates Virus Replication by Targeting MAVS. Vet. Microbiol..

[78] Zhang D., Tang Z., Huang H., Zhou G., Cui C., Weng Y., Liu W., Kim S., Lee S., Perez-Neut M. (2019). Metabolic Regulation of Gene Expression by Histone Lactylation. Nature.

[79] Pang Y., Zhou Y., Wang Y., Fang L., Xiao S. (2024). Lactate-Lactylation-HSPA6 Axis Promotes PRRSV Replication by Impairing IFN-β Production. J. Virol..

[80] Takamatsu Y., Yoshikawa T., Kurosu T., Fukushi S., Nagata N., Shimojima M., Ebihara H., Saijo M., Noda T. (2022). Role of VP30 Phosphorylation in Ebola Virus Nucleocapsid Assembly and Transport. J. Virol..

[81] Chen X., Wang X. (2024). The HIV-1 Gag P6: A Promising Target for Therapeutic Intervention. Retrovirology.

[82] Liu Q., Wang H., Zhang H., Sui L., Li L., Xu W., Du S., Hao P., Jiang Y., Chen J. (2022). The Global Succinylation of SARS-CoV-2-Infected Host Cells Reveals Drug Targets. Proc. Natl. Acad. Sci. USA.

[83] Chen X., Wang S., Wu M., Zhao Y. (2023). Role of Succinylation in Pseudorabies Virus Infection. J. Virol..

[84] Chen X., Wang S., Chen K., Han Q. (2024). The Global Landscapes of Lysine Crotonylation in Pseudorabies Virus Infection. Virology.

